# Identifying and handling data bias within primary healthcare data using synthetic data generators

**DOI:** 10.1016/j.heliyon.2024.e24164

**Published:** 2024-01-10

**Authors:** Barbara Draghi, Zhenchen Wang, Puja Myles, Allan Tucker

**Affiliations:** a Medicines and Healthcare products Regulatory Agency, London, UK; b Brunel University London, London, UK

**Keywords:** Synthetic data generators, Data bias, Over-sampling, Bayesian networks, Machine learning

## Abstract

Advanced synthetic data generators can simulate data samples that closely resemble sensitive personal datasets while significantly reducing the risk of individual identification. The use of these advanced generators holds enormous potential in the medical field, as it allows for the simulation and sharing of sensitive patient data. This enables the development and rigorous validation of novel AI technologies for accurate diagnosis and efficient disease management. Despite the availability of massive ground truth datasets (such as UK-NHS databases that contain millions of patient records), the risk of biases being carried over to data generators still exists. These biases may arise from the under-representation of specific patient cohorts due to cultural sensitivities within certain communities or standardised data collection procedures. Machine learning models can exhibit bias in various forms, including the under-representation of certain groups in the data. This can lead to missing data and inaccurate correlations and distributions, which may also be reflected in synthetic data. Our paper aims to improve synthetic data generators by introducing probabilistic approaches to first detect difficult-to-predict data samples in ground truth data and then boost them when applying the generator. In addition, we explore strategies to generate synthetic data that can reduce bias and, at the same time, improve the performance of predictive models.

## 1 Introduction

The use of synthetic data in healthcare is a promising solution to the challenges of developing AI systems while protecting patient privacy, which has been a significant concern under the General Data Protection Regulation [1]. Synthetic data generation is an effective technique that enables the capture of structure and distributions found in actual data sets, all while safeguarding patient privacy and mitigating the risks of individual identification. One way of achieving this is through the use of generative models built based on real data [2]. These models can either be hand-coded through expert knowledge or inferred from real data using models such as Bayesian networks (BNs) [3]. Once created, they can generate synthetic data by using sampling techniques. Despite using well-established techniques such as Bayesian networks for generating high-fidelity synthetic patient data [4], and despite access to huge datasets, biases may persist and be propagated through the data generation process. The presence of biases within data has become a significant issue in implementing AI techniques. Indeed, replicating and even amplifying human biases, particularly those affecting protected groups, is a significant risk [5]. Algorithmic bias manifests in various ways, with varying degrees of impact on affected groups. For example, biases may arise in online recruitment tools [6], word association [7], and criminal justice decision-making [8] among others [9]. Biased training data has caused machine learning models to make biased decisions, as pointed out in several studies ([10], [11]). This is because selecting data from a biased population sample leads to decisions that reflect the biases already present in our society. Focusing on the domain of healthcare and biomedical fields, a longstanding history of discrimination in medicine can be discovered [12] [13] [14]. Demographic healthcare inequalities persist worldwide, and the impact of medical biases on different patient groups is still an issue. In this scenario, AI represents an excellent opportunity to handle bias-related issues. However, there are several examples of how the lack of bias detection systems is an issue that most of the currently used biomedical AI technologies present. Gender and sex bias can have a significant impact on precision medicine [15], as well as bias can be a problem when applying machine learning approaches to outcome prediction in anticoagulant drug therapy [16]. As previously discussed, machine learning bias can manifest in various ways [17]. While entirely eradicating bias from our society may not be feasible, we can implement strategies to eliminate bias from our data and models. In this study, bias refers to the under-representation of specific patient groups, regardless of the cause. Synthetic data generated from biased data can lead to the under-representation of certain patient groups due to cultural sensitivities amongst some communities or standardised procedures in data collection. This may result in missing or incorrect correlations and distributions that reflect the biases present in the ground truth datasets. Datasets in medicine are often imbalanced, and the under-representation of specific patient groups reflects this bias. There are different approaches to address imbalances in data and mitigate bias. Some of these approaches are de-biasing methods, such as Reweighing [18] [19], Adversarial Debiasing [20], Reject option classification [21] [22], Equality of Opportunity [23] and Prejudice Remover Regularizer [21]. Other methods include the generation of synthetic data, including SMOTE [24] and variants such as Adaptive Synthetic Sampling (AdaSyn) [25]. The de-biasing methods aim to mitigate the bias in the training data to create an unbiased model when making decisions based on specific sensitive attributes. In contrast, SMOTE and AdaSyn re-balance the data considering the class variables. Although helpful in mitigating bias, these tools degrade learner performance as a side effect of improving fairness. Achieving fairness and high performance simultaneously is an ambitious goal defined as impossible in the past [26]. However, state-of-the-art bias mitigation algorithms, including Fair-SMOTE [27], addressed this challenge. Fair-SMOTE balances data based on class and sensitive attributes such that privileged and unprivileged groups have equal positive and negative examples. While it is beneficial when the protected attribute is binary, Fair-SMOTE has some limitations when it comes to achieving our specific purposes. Given that our goal is to identify different groups subject to bias, dividing the population into privileged and unprivileged a priori would cause a significant loss of information to identify specific cohorts of patients. However, since FAIR-SMOTE represents the state-of-the-art bias mitigation approaches, comparisons with its application are also proposed in this work with the necessary simplifications. This paper explores BayesBoost, a technique that combines a Bayesian network synthetic data generator with a boosting approach. The primary objective of this method is to detect under-represented samples in a dataset and subsequently use the synthetic data to over-sample the under-represented groups, resulting in a better distribution of overall features. This work extends our preliminary research approach [28], published as a conference paper. Despite some common ground with the previous conference publication, the innovation introduced in this new framework relies on refinement, improvement and optimisation of the methodology, including changes and enhancements within the bias detection framework, by introducing a stratified sampling in the so-called uncertainty analysis, but also in the bias correction framework, by optimising the Bayesian network application. Moreover, a complete and extended evaluation has been carried out, investigating several diseases and several protected attributes which better refer to minority groups, including ethnicity. Nevertheless, a thorough comparison with the state-of-the-art approach, Fair-SMOTE, is proposed in this work. Our work differs from the existing techniques mentioned above since it aims to create synthetic data that is more representative of the entire population, thus enhancing the performance of predictive models. The rest of the paper is organised as follows. Section 2 provides a detailed methodology definition, introducing BayesBoost and explaining our simulation approach to simulate data biases. After presenting the data used for testing the method, empirical analysis is proposed. Section 3 offers the results obtained from the application of BayesBoost, and finally, the conclusion is described in section 4.

## 2 Method

The approach we propose aims to identify data biases, correct them and improve classification accuracy. In this section, we define the developed methodology in detail. Firstly, we provide a comprehensive explanation of BayesBoost. We then describe the data bias simulation approach used to generate biased data to evaluate the effectiveness of our methodology. Next, we introduce the datasets on which we test our method. Finally, we describe the empirical analysis.

### 2.1 Methodology

*BayesBoost*  BayesBoost can be broken down into two main sections. First, an uncertainty analysis is carried out for identifying data biases. In order to identify groups of under-represented data, the idea is to test a classifier, trained on a dataset, in predicting a binary target on a validation set extracted a priori from data. A disease target and a protected attribute are selected. The protected attribute is an attribute that divides the population into several groups within which we want to investigate under-representations. After choosing the protected attribute, a validation set is extracted through stratified random sampling based on that attribute's levels. Extracting a validation set through stratified random sampling avoids obtaining results biased from the original data distribution. In order to detect under-represented data groups, we analyse the performance of a chosen classifier in predicting the target disease. Specifically, we define all the subjects where our classifier shows uncertainty in the prediction as difficult to classify. We determine uncertainty through probabilities that fall within designated intervals, with probabilities ranging from 0.4 to 0.7 indicating uncertain binary classifications. We used an iterative approach and quartile-based analysis to determine p1 and p2, the uncertainty probabilities for our binary classification model. Our first probability, p1 = 0.4, represents the average probability of being classified as the negative class (0) within the 0.25-0.5 quartile range. The second probability, p2 = 0.7, represents the average probability of uncertainty for cases with a probability range between 0.5 and 0.75 for being classified as the positive class (1). This iterative process involved ten repeated classifications and the extraction of average values to ensure that these probabilities accurately reflect uncertainty levels in our dataset. The data classified with uncertainty within this interval forms a new dataset called D_Unc_. Although any binary classification model can potentially be used for uncertainty analysis, we experimented with a Naïve Bayes classifier due to its simplicity and probabilistic nature. Another important thing to note is that when performing the uncertainty analysis, it is essential to utilise appropriate metrics. Since we aim to improve the representativeness of data but also the effectiveness of predictive models, we use classification accuracy as the metric to extract under-represented groups.

The second section of this work concerns the application of a synthetic data generator to overcome the biases highlighted through the uncertainty analysis. Attributes are sorted based on differences between the distributions of the data we are investigating for bias, referred to D_Bias_, and D_Unc_, framing the ordered set of variables named O. The idea is to generate a set of *m* rows for each D_Unc_ row, utilising a Bayesian network trained on D_Bias_. The network incorporates evidence from D_Unc_, thus including subjects with under-represented characteristics in D_Bias_ to generate fresh data samples. The resulting synthetic dataset is merged with D_Bias_ to produce the ultimate dataset, *BB*. In our approach, the number of rows to extract *m* is an additional parameter that may need future optimisation. We attempted three different methods. The first method involves extracting *m* data for each row of D_Unc_ to create a dataset that is half the size of D_Bias_. The second method involves extracting *m* data to generate a dataset with dimensions equal to D_Bias_. The third method involves extracting *m* rows for each row of D_Unc_ in such a way as to create a dataset with dimensions that are twice that of D_Bias_. The complete details of this entire process are fully documented in Algorithm 1.

**Algorithm 1:**
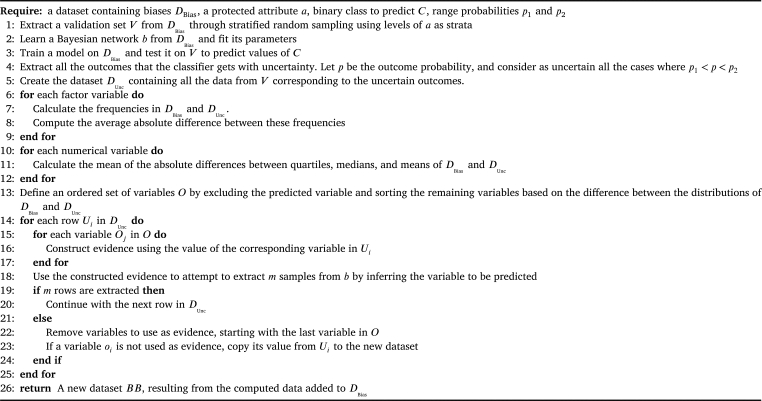
BayesBoost Pseudocode.

*Simulation of data bias*  Two experiments are proposed within this work. One experiment presents the application of BayesBoost directly on the ground truth data. However, first, a simulation experiment is proposed to test the efficacy of BayesBoost. Given a dataset, data bias is simulated by generating synthetic data containing biases. Applying our boosting method to a simulated data set is necessary to show its functioning and effectiveness. Knowing the biases (because we introduce them) allows seeing if the developed approach works. Bayesian networks (BNs) are used to simulate biased data, thanks to their intrinsic properties. BNs are probabilistic models representing a set of stochastic variables with their respective dependencies and conditional distributions. Hence, they enable the generation of random samples under particular evidence, enabling the creation of data with predetermined biases that can be useful in testing our approach. After learning a Bayesian network from the original data, the protected attribute to investigate is selected. New conditional probabilities are introduced for categorical variables within the Bayesian network from which the data will be extracted. Finally, the percentage of data to be under-represented is identified. For instance, if 30% is chosen, a data set containing 30% of subjects with the chosen characteristic will be generated. After selecting these parameters, the data is extracted from the Bayesian network using logic sampling [29]. Controlling the level of under-sampled cases when generating synthetic data can be achieved by using evidence to produce data with the exact degree of under-sampling required. In order to completely separate the biased data from the original data, a Bayesian network is learned from the obtained dataset. A dataset of the desired size, which from now on we will refer to as D_Bias_, is extracted from this network. In our simulation, D_Bias_ is the data set that represents the original data set on which to apply the method for identifying and correcting data biases.

### 2.2 Datasets

The developed approach is tested on synthetic datasets generated from anonymised real primary care data [30] from the Clinical Practice Research Datalink (CPRD). CPRD is a real-world research service supporting retrospective and prospective public health and clinical studies in the UK. It is jointly sponsored by the Medicines and Healthcare products Regulatory Agency and the National Institute for Health Research, as part of the Department of Health and Social Care [31]. First, the approach is applied to the CPRD Synthetic cardiovascular disease datasets (CVD) [32], a dataset focusing on cardiovascular disease risk factors. The dataset covers 499,344 patients and 21 variables, including stroke or heart attack, smoking habits, region, age, chronic diseases, body mass index, systolic blood pressure and other cardiovascular disease risk factors. CVD is a mixed dataset because it contains both numeric and factor variables. Finally, the method is applied to the CPRD Covid-19 Synthetic datasets [33], which focuses on patients presenting to primary care with symptoms indicative of Covid-19 (confirmed/suspected Covid-19) and control patients with negative Covid-19 test results. The dataset covers 779,546 patients and 47 variables, including age, age categories, gender, region, Covid-19 diagnosis and Covid-19 test results. Even though the datasets we mentioned are not real, they closely resemble real-world primary healthcare data in terms of key characteristics and patterns, as demonstrated by studies [4]. Due to their high fidelity, we opted to test our approach on these synthetic datasets to avoid any privacy concerns that were previously explained.

### 2.3 Experimental design

The experiments that are carried-out can be divided into two subgroups: simulation and direct application. When conducting a simulation experiment (where we artificially create under representations to test our approach), an additional step for generating synthetic data containing biases is proposed. Our study uses the above-described datasets, and for each dataset, several targets and protected attributes are investigated. Regarding the CVD synthetic dataset, stroke and heart attacks, atrial fibrillation and type two diabetes are considered disease targets, whilst ethnicity, region and gender are investigated as biases.

Considering the Covid-19 synthetic dataset, the Covid-19 diagnosis is the target while gender, age categories and region are considered protected attributes. For every experiment, we split the datasets (train - 70%, test – 30%) and select the target and protected attribute we want to investigate. A dataset containing forced biases is generated using a synthetic data generator when conducting a simulation experiment. Otherwise, 70% of original data are carried on as data containing biases. Remember that the simulation experiment was initially necessary to assess our approach's efficacy, but in real work, we should follow the path of the experiment where we don't need simulation of biases. The dataset is then split again into a train and validation set. The validation set is extracted via stratified random sampling using the protected attribute's levels as strata. After building a probabilistic model on the train data, we test the classifier in predicting the binary target on the validation set. BayesBoost is applied to detect under-represented groups.

First, the uncertainty analysis is carried out to identify the under-represented groups. Second, the synthetic data generator is applied to boost the uncertain cases. For each attempt, the results of BayesBoost are three synthetic datasets: BB_50_, BB_100_ and BB_200_, depending on the degree of oversampling used in the BayesBoost Algorithm. BB_50_ results from the extraction of *m* data to boost the original data with an extra 50% of the size of D_Bias_. BB_100_ results from the extraction of m data to boost the dataset by 100%. BB_200_ is the outcome when extracting m data to boost the dataset by 200%. In order to assess the efficacy of our approach, when conducting both types of experiments, we generate synthetic datasets by applying SMOTE and Adaptive Synthetic Sampling (AdaSyn) to the dataset in which biases have been deliberately introduced. Then, we compare the two outcomes to those obtained by BayesBoost. Moreover, when conducting the direct application experiment, BayesBoost is compared to the start of art approach Fair-SMOTE using the following assumptions:

• when investigating gender as a protected attribute, males are considered privileged
• when investigating ethnicity as a protected attribute, “White or not stated” and “Other ethnic groups” are considered privileged. The remaining eight groups are considered unprivileged
• when investigating region as a protected attribute, “London” and “South Central” are considered privileged. The remaining eight groups are considered unprivileged

**Figure 1:**
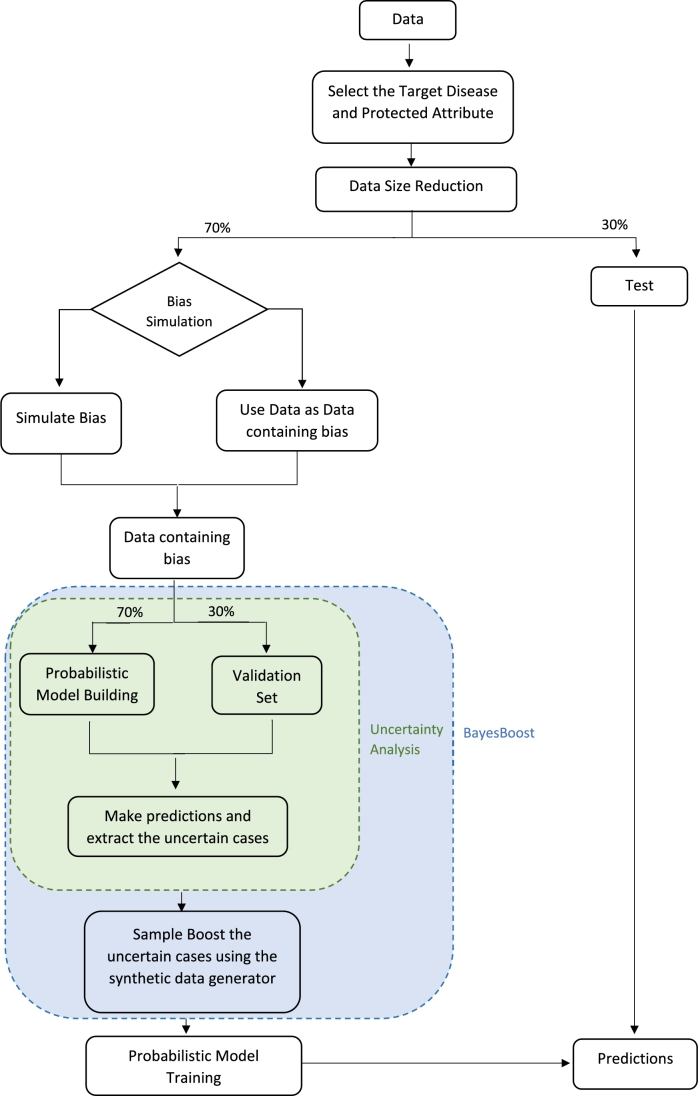
Block diagram of BayesBoost.

Dividing data into privileged and unprivileged may limit acquiring data that accurately represents the entire population. This process results in losing all non-binary protected-attribute information for each group, making it challenging to identify specific patient cohorts. Despite the potential limitations, the resulting synthetic data sets are compared in predicting a binary variable by training a Naive Bayes classifier and testing the models on the same independent test set. We report the mean of ten runs and confidence intervals from applying the t-test. Fig. 1 shows the block diagram for one repeat of our experiment.

## 3 Results

This section proposes the results obtained from the application of BayesBoost. We choose to show results that better helps to answer three essential research questions. More results are available at Appendix A.

### 3.1 Simulation experiments

**Figure 2:**
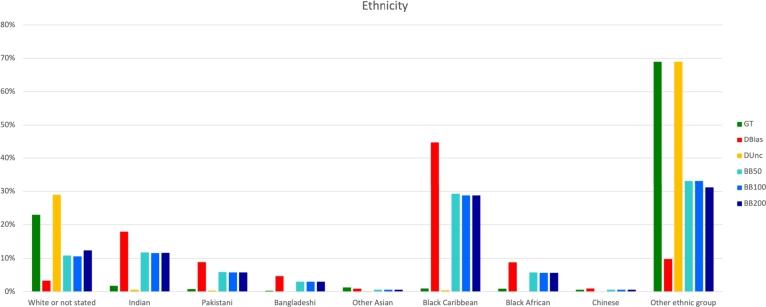
Ethnicity distribution obtained by simulation experiment on CVD data.

*BayesBoost: identification of data bias*  The first research question is: can BayesBoost highlight data bias? To answer this question, we can consider Fig. 2, which shows the results obtained from a simulation experiment conducted on the CVD dataset where “ethnicity” is the protected attribute and stroke and heart attacks are the target disease. In Fig. 2, green bars represent the spread distribution of ethnicity in the ground truth data, whilst yellow bars represent the uncertainty analysis outcomes. Red bars represent the distribution of ethnicity in the simulated data where we purposely reduce “White or not stated” and “Other ethnic group” whilst increasing the others. When analysing simulation experiment results, yellow bars must be compared to the red ones, which represent data containing bias. The idea is that if the yellow bar is higher than the red bar, it highlights that we need more cases of these groups. As we can see in Fig. 2, BayesBoost manages to identify the under-representation we introduced, as we can see from the yellow bars that tell us that we need more “White or not stated” and “Other ethnic group”. Also, yellow bars tell us that we don't need more Indian, Pakistani, Bangladeshi, Other Asian, the Black Caribbean, Black African and Chinese.

*BayesBoost: data bias correction*  The second research question is: can BayesBoost correct data bias? In order to answer this question, we can refer to the same results we used to answer the previous research question. In Fig. 2, blue bars represent the distribution of ethnicity in the datasets resulting from the application of the boosting approach. Therefore, they represent the dataset corrected for bias. Comparing blue bars to red ones in Fig. 2 shows that our approach is working. For example, if we consider the “White or not stated” group, the red bar represents the simulation, and the yellow bar tells us that we need more subjects belonging to this category. The blue bar shows an increase of the subjects belonging to this category, as can be seen by comparing the red and blue bars.

*BayesBoost: classification accuracy improvement*  The third research question is: can BayesBoost improve classification accuracy? Our approach aims not only to generate synthetic data where biases have been reduced but also to obtain better predictive models for desired diseases. Table 1 summarised the classification accuracy and respective confidence intervals for the simulation experiment. Also, AUC values calculated for the ROC and precision-recall curves are proposed. We chose to use the AUC as a metric since it represents a valid measure of classification performance [34]. The results contained in Table 1 allow us to be sure that the method works. The classification accuracy obtained by testing the D_Bias_ dataset decreases significantly, which means that the synthetic generation of bias data has been successful. Furthermore, we can see how the application of BayesBoost leads to increasing classification performance. AUC values computed for ROC and Precision-Recall curves are comparable among BayesBoost, Smote and AdaSyn.

**Table 1:** Results obtain by simulation experiments. D_Bias_ is obtained through generation of synthetic data containing bias.

Data	Protected	Target	Dataset	CI low	Classification	CI up	AUC	AUC
	Attribute				Accuracy		ROC	P-R
CVD	Ethnicity	Stroke	D	0.79	0.792	0.793	0.85	0.32
			D_Bias_	0.596	0.597	0.598	0.78	0.25
			BB50	0.751	0.753	0.754	0.8	0.28
			BB100	0.752	0.753	0.754	0.8	0.29
			BB200	0.758	0.754	0.756	0.8	0.28
			SMOTE	0.714	0.715	0.716	0.81	0.29
			AdaSyn	0.68	0.69	0.692	0.8	0.28

CVD	Ethnicity	Atrial Fibrillation	D	0.892	0.893	0.894	0.87	0.16
			D_Bias_	0.69	0.7	0.71	0.76	0.15
			BB50	0.831	0.83	0.84	0.87	0.16
			BB100	0.842	0.843	0.845	0.87	0.16
			BB200	0.855	0.856	0.857	0.87	0.16
			SMOTE	0.742	0.745	0.748	0.87	0.16
			AdaSyn	0.74	0.742	0.745	0.87	0.16

CVD	Ethnicity	Type 2 Diabetes	D	0.81	0.82	0.823	0.84	0.25
			D_Bias_	0.7	0.71	0.724	0.8	0.21
			BB50	0.791	0.793	0.796	0.82	0.22
			BB100	0.796	0.797	0.798	0.82	0.23
			BB200	0.797	0.798	0.8	0.83	0.23
			SMOTE	0.701	0.705	0.71	0.82	0.22
			AdaSyn	0.692	0.694	0.698	0.81	0.23

### 3.2 Real data experiments

**Figure 3:**
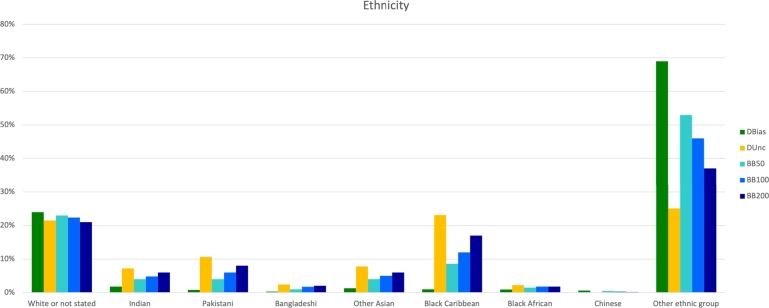
Ethnicity distribution obtained by direct application experiment on CVD data.

**Figure 4:**
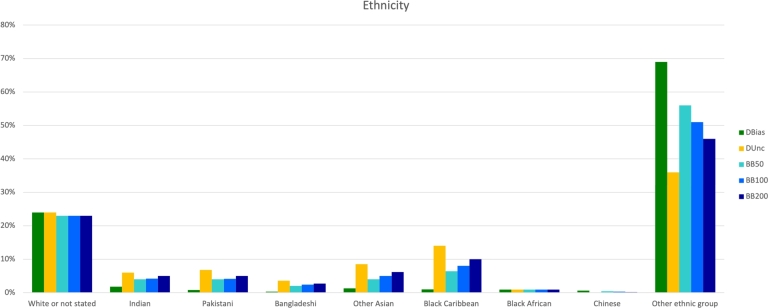
Ethnicity distribution obtained by direct application experiment on CVD data when predicting atrial fibrillation.

**Figure 5:**
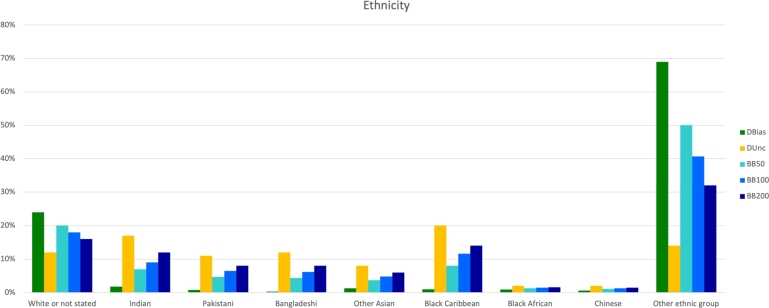
Ethnicity distribution obtained by direct application experiment on CVD data when predicting type 2 diabetes.

**Figure 6:**
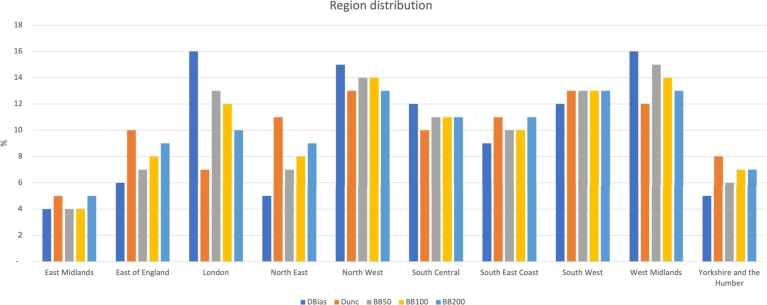
Region distribution obtained by direct application experiment on CVD data when predicting stroke.

**Figure 7:**
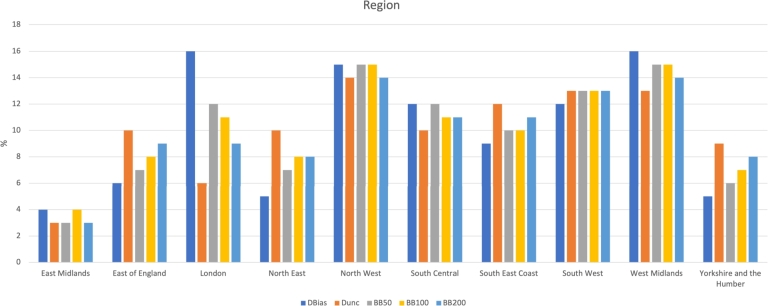
Region distribution obtained by direct application experiment on CVD data when predicting atrial fibrillation.

**Figure 8:**
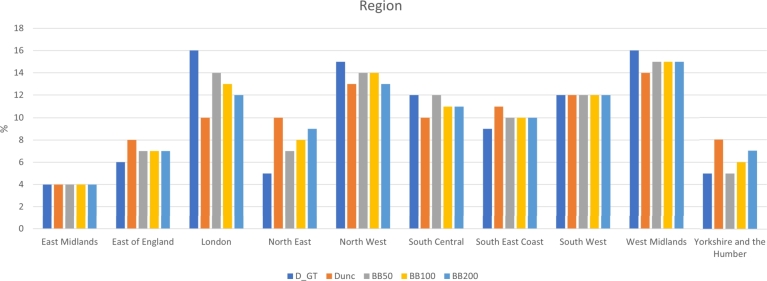
Region distribution obtained by direct application experiment on CVD data when predicting type 3 diabetes.

**Figure 9:**
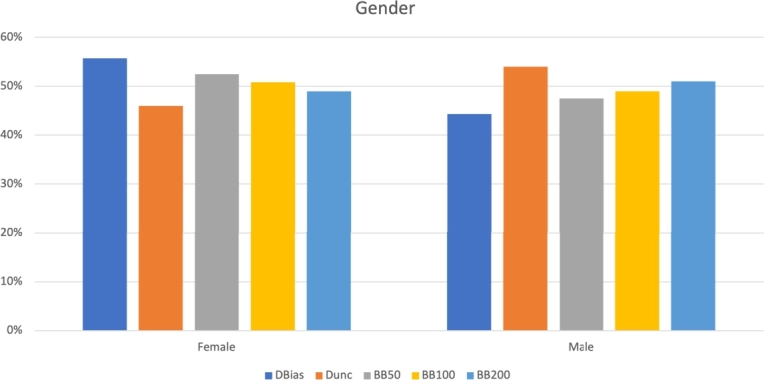
Gender distribution obtained by direct application experiment on CVD data when predicting stroke.

**Figure 10:**
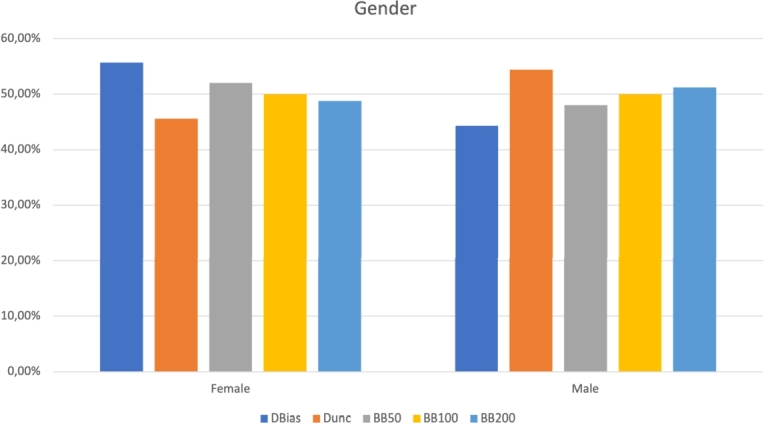
Gender distribution obtained by direct application experiment on CVD data when predicting atrial fibrillation.

**Figure 11:**
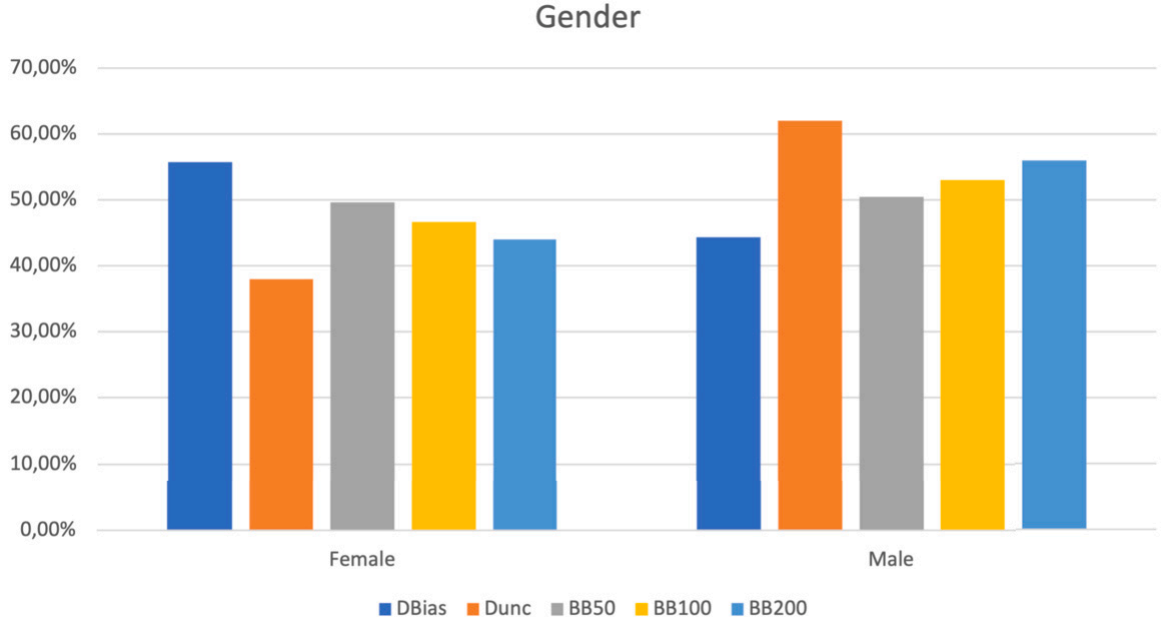
Gender distribution obtained by direct application experiment on CVD data when predicting type 2 diabetes.

**Figure 12:**
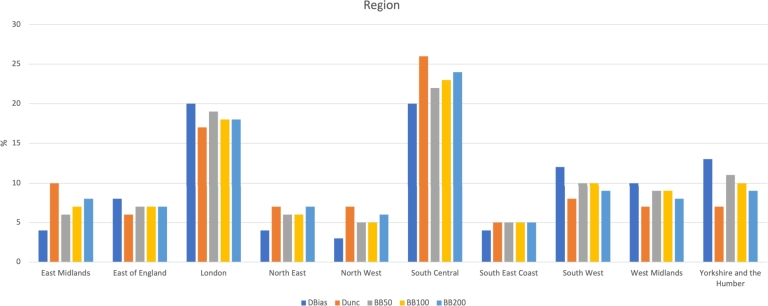
Region distribution obtained by direct application experiment on Covid-19 data when predicting Covid-19 diagnosis.

**Figure 13:**
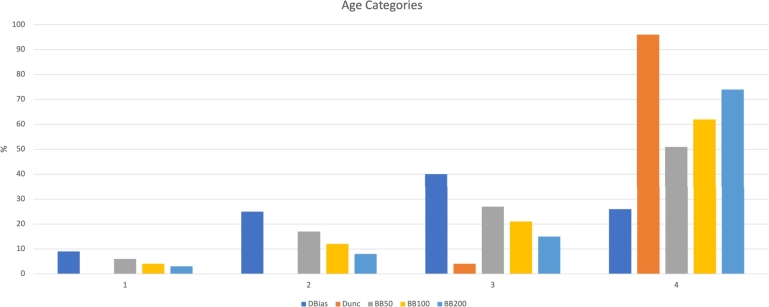
Age categories distribution obtained by direct application experiment on Covid-19 data when predicting Covid-19 diagnosis. Age category “1” represents patients under 20, and age category “2” patients aged between 20 and 44. Age category 3 corresponds to patients aged 45 to 69, and “4” is assigned to patients over 70.

*BayesBoost: identification of data bias*  To answer this question, we can consider Fig. 3, which shows the results obtained from a direct application experiment conducted on the CVD dataset where “ethnicity” is the protected attribute and stroke and heart attacks are the target disease. In Fig. 3, green bars represent the spread distribution of ethnicity in the ground truth data, whilst yellow bars represent the uncertainty analysis outcomes. When analysing the direct application experiment, yellow bars must be compared to the green ones, which represent data containing bias. The idea is that if the yellow bar is higher than the green bar, it highlights that we need more cases of these groups. Results showed in Fig. 3 highlight that we need more Indian, Pakistani, Bangladeshi, Other Asian, Black Caribbean and Black African if we want to obtain a fairer dataset and a better model to predict stroke and heart attacks. Also, “White or not stated” and “Other ethnic group” groups don't need to be increased. It means that the original data already contains enough of them for our purpose. Data distributions resulting from additional conducted real data experiments can be found in Appendix A. Fig. 4 in the appendix reports ethnicity distribution when investigating racial biases in predicting atrial fibrillation, while Fig. 5 displays the same for predicting type 2 diabetes. Figure 6, Figure 7, Figure 8 refer to investigating regional biases in predicting stroke and heart attacks, type 2 diabetes, and atrial fibrillation, respectively. Whereas Figure 9, Figure 10, Figure 11 delve into the gender biases in predicting stroke and heart attacks, type 2 diabetes, and atrial fibrillation, respectively. Additionally, Fig. 12 outlines the regional distribution obtained while investigating biases in Covid-19 data. Fig. 13 presents the distribution of age categories when examining age biases in predicting Covid-19 diagnosis.

*BayesBoost: data bias correction*  The second research question is: can BayesBoost correct data bias? In order to answer this question, we can refer to the same results we used to answer the previous research question. In Fig. 3, blue bars represent the distribution of ethnicity in the datasets resulting from the application of the boosting approach. Therefore, they represent the dataset corrected for bias. Comparing blue bars to green ones in Fig. 3 show that our approach is working. For example, if we consider the “Indian” group, the green bar represents the ground truth data, and the yellow bar tells us that we need more subjects belonging to this category. Blue bars increase the number of subjects belonging to this category, as seen by comparing the green and blue bars.

*BayesBoost: classification accuracy improvement*  The third research question is: can BayesBoost improve classification accuracy? Our approach aims not only to generate synthetic data where biases have been reduced but also to obtain better predictive models for desired diseases. Table 2, Table 3 contain three performance metrics for every experiment. Prediction performance is measured in terms of classification accuracy, AUC values calculated for the ROC and precision-recall curves. In Table 2, Table 3 the performances obtained with BayesBoost, SMOTE and Adasyn and F-SMOTE are compared. As we can see, BayesBoost always increases classification accuracy while also maintaining reasonable confidence intervals. When applying SMOTE, AdaSyn and F-SMOTE classification accuracy drops. Moreover, SMOTE and AdaSyn solve the class imbalanced issue but do not mitigate bias related to protected attributes. F-SMOTE, which aims to mitigate bias while increasing classification accuracy, unlikely SMOTE and AdaSyn, shows a lower classification accuracy than that obtained by the application of BayesBoost. However, when applying F-Smote, AUC computed for the precision-recall curve increases. That's because F-Smote, mitigating bias while rebalancing the classification problem, increases the Recall with the side effect of decreasing classification accuracy and AUC calculated for the ROC curve. Moreover, to apply F-SMOTE, we had to make assumptions and simplifications, as explained in section 2.3. Consequently, all non-binary protected-attributes information about each group is lost as all data is divided into privileged and unprivileged. Furthermore, considering the final distributions of the data, we have seen how the application of BayesBoost leads to rebalancing data by both increasing and decreasing data groups. F-SMOTE instead leads to having the same number of cases within each group (privileged and class 0, privileged and class 1, unprivileged and class 0, non-privileged and class 1). Obtaining such a distribution of data may not be representative of reality. BayesBoost aims to mitigate the data bias by generating synthetic data that are more representative of the ground truth data population while improving performance. As we have seen from Figure 2, Figure 3, BayesBoost does not lead to having the same number of cases in each level but rebalances the data to have a faithful representation of reality, which can involve a decrease in the original cases.

**Table 2:** Results obtain by direct application experiment. D_Bias_ represent the original data that we supposed to contain bias.

Data	Protected	Target	Dataset	CI low	Classification	CI up	AUC	AUC
	Attribute				Accuracy		ROC	P-R
CVD	Ethnicity	Stroke	D_Bias_	0.793	0.795	0.796	0.741	0.37
			BB50	0.824	0.826	0.827	0.746	0.37
			BB100	0.827	0.829	0.83	0.74	0.37
			BB200	0.828	0.83	0.831	0.73	0.36
			SMOTE	0.715	0.717	0.718	0.73	0.37
			AdaSyn	0.687	0.688	0.69	0.73	0.37
			F-SMOTE	-	0.65	-	0.63	0.4

CVD	Ethnicity	Atrial Fibrillation	D_Bias_	0.887	0.888	0.889	0.8	0.19
			BB50	0.927	0.928	0.929	0.81	0.18
			BB100	0.934	0.935	0.936	0.81	0.18
			BB200	0.94	0.941	0.942	0.79	0.18
			SMOTE	0.744	0.746	0.748	0.79	0.18
			AdaSyn	0.741	0.742	0.744	0.79	0.18
			F-SMOTE	-	0.71	-	0.7	0.4

CVD	Ethnicity	Type 2 Diabetes	D_Bias_	0.821	0.823	0.824	0.712	0.306
			BB50	0.838	0.84	0.841	0.714	0.323
			BB100	0.843	0.844	0.846	0.711	0.331
			BB200	0.845	0.846	0.847	0.711	0.341
			SMOTE	0.742	0.744	0.745	0.74	0.384
			AdaSyn	0.707	0.709	0.711	0.73	0.366
			F-SMOTE	-	0.66	-	0.59	0.38

CVD	Region	Stroke	D_Bias_	0.793	0.795	0.796	0.745	0.367
			BB50	0.817	0.818	0.819	0.741	0.372
			BB100	0.823	0.824	0.826	0.739	0.369
			BB200	0.829	0.83	0.831	0.731	0.36
			SMOTE	0.714	0.715	0.716	0.739	0.369
			AdaSyn	0.688	0.69	0.692	0.74	0.371
			F-SMOTE	-	0.66	-	0.63	0.44

CVD	Region	Atrial Fibrillation	D_Bias_	0.889	0.889	0.89	0.807	0.187
			BB50	0.92	0.922	0.923	0.807	0.194
			BB100	0.930	0.931	0.932	0.802	0.19
			BB200	0.941	0.942	0.944	0.802	0.186
			SMOTE	0.747	0.749	0.751	0.802	0.187
			AdaSyn	0.746	0.747	0.749	0.797	0.184
			F-SMOTE	-	0.79	-	0.72	0.42

CVD	Region	Type 2 Diabetes	D_Bias_	0.822	0.823	0.824	0.712	0.308
			BB50	0.836	0.837	0.838	0.7	0.307
			BB100	0.838	0.84	0.842	0.697	0.316
			BB200	0.842	0.844	0.845	0.7	0.333
			SMOTE	0.739	0.74	0.742	0.732	0.374
			AdaSyn	0.702	0.703	0.705	0.724	359
			F-SMOTE	-	0.67	-	0.62	0.41

**Table 3:** Results obtain by direct application experiment. D_Bias_ represent the original data that we supposed to contain bias.

Data	Protected	Target	Dataset	CI low	Classification	CI up	AUC	AUC
	Attribute				Accuracy		ROC	P-R
CVD	Gender	Stroke	D_Bias_	0.793	0.795	0.796	0.741	0.367
			BB50	0.817	0.818	0.819	0.74	0.371
			BB100	0.823	0.824	0.826	0.736	0.371
			BB200	0.829	0.83	0.831	0.734	0.367
			SMOTE	0.714	0.715	0.716	0.738	0.376
			AdaSyn	0.688	0.69	0.692	0.738	0.372
			F-SMOTE	-	0.63	-	0.64	0.45

CVD	Gender	Atrial Fibrillation	D_Bias_	0.889	0.889	0.89	0.807	0.186
			BB50	0.92	0.922	0.92	0.801	0.185
			BB100	0.930	0.931	0.932	0.8	0.184
			BB200	0.941	0.942	0.944	0.792	0.175
			SMOTE	0.747	0.749	0.751	0.796	0.178
			AdaSyn	0.746	0.747	0.749	0.798	0.184
			F-SMOTE	-	0.78	-	0.72	0.42

CVD	Gender	Type 2 Diabetes	D_Bias_	0.822	0.82	0.823	0.71	0.299
			BB50	0.836	0.84	0.841	0.703	0.308
			BB100	0.838	0.84	0.842	0.703	0.317
			BB200	0.842	0.844	0.845	0.703	0.327
			SMOTE	0.739	0.74	0.742	0.729	0.365
			AdaSyn	0.702	0.703	0.705	0.723	0.353
			F-SMOTE	-	0.6	-	0.51	0.35

Covid-19	Age Categories	Covid Diagnosis	D_Bias_	0.916	0.917	0.919	0.831	0.218
			BB50	921	0.922	0.923	0.831	0.214
			BB100	0.926	0.928	0.929	0.827	0.212
			BB200	0.932	0.933	0.934	0.827	0.213
			SMOTE	0.705	0.706	0.707	0.819	0.207
			AdaSyn	0.719	0.721	0.722	0.828	0.22
			F-SMOTE	-	0.6	-	0.6	0.38

Covid-19	Region	Covid Diagnosis	D_Bias_	0.918	0.919	0.92	0.831	0.214
			BB50	0.918	0.92	0.921	0.825	0.209
			BB100	0.926	0.928	0.929	0.826	0.214
			BB200	0.932	0.933	0.934	0.824	0.215
			SMOTE	0.712	0.714	0.717	0.819	0.215
			AdaSyn	0.733	0.735	0.737	0.828	0.221
			F-SMOTE	-	0.66	-	0.58	0.35

Covid-19	Gender	Covid Diagnosis	D_Bias_	0.918	0.919	0.92	0.834	0.221
			BB50	0.918	0.92	0.922	0.832	0.219
			BB100	0.926	0.928	0.929	0.834	0.224
			BB200	0.932	0.933	0.934	0.834	0.224
			SMOTE	0.712	0.714	0.717	0.824	0.218
			AdaSyn	0.733	0.735	0.737	0.831	0.22
			F-SMOTE	-	0.69	-	0.58	0.34

### 3.3 Extending beyond accuracy to other metrics

Although the initial focus of BayesBoost was on predictive accuracy, the approach has the potential to facilitate other metrics. To explore this potential, we conducted some initial experiments to assess how well the method generalizes to other metrics, particularly fairness metrics like Equalized Odds and Demographic Parity. Our observations indicated that changing the criteria for selecting uncertain cases led to improvements in relevant statistics (see Table 4, Table 5) but did not always guarantee an improvement in underlying fairness metrics across the board. This was likely due to the interactive nature of these metrics, with some showing promise while others required fine-tuning during resampling.

**Table 4:** Performance metrics with equalised odds as a metric for uncertainty analysis in CVD Data with Ethnicity as the protected attribute and stroke as the target disease.

Metric	DBias	BB50	BB100	BB200
Accuracy	0.905	0.882	0.832	0.731
Accuracy 95% CI	0.904, 0.907	0.881, 0.882	0.83, 0.833	0.729, 0.732
Precision	0.372	0.312	0.254	0.202
Precision 95% CI	0.365, 0.379	0.309, 0.315	0.251, 0.256	0.2, 0.204
Recall	0.302	0.409	0.584	0.823
Recall 95% CI	0.296, 0.308	0.404, 0.414	0.578, 0.59	0.819, 0.827
F1-Score	0.333	0.354	0.354	0.324
F1-Score 95% CI	0.327, 0.339	0.35, 0.358	0.351, 0.356	0.322, 0.327

**Table 5:** Performance metrics with demographic parity as a metric for uncertainty analysis in CVD Data with Ethnicity as the protected attribute and stroke as the target disease.

Metric	DBias	BB50	BB100	BB200
Accuracy	0.904	0.882	0.853	0.728
Accuracy 95% CI	0.903, 0.905	0.881, 0.883	0.851, 0.854	0.727, 0.731
Precision	0.388	0.326	0.284	0.209
Precision 95% CI	0.381, 0.395	0.319, 0.333	0.28, 0.288	0.205, 0.213
Recall	0.298	0.407	0.519	0.83
Recall 95% CI	0.289, 0.307	0.399, 0.415	0.512, 0.526	0.827, 0.833
F1-Score	0.337	0.361	0.368	0.332
F1-Score 95% CI	0.329, 0.345	0.354, 0.368	0.365, 0.371	0.326, 0.338

## 4 Conclusion

Detecting underrepresented groups of patients is a valuable approach, particularly when it comes to generating synthetic data. BayesBoost is an effective technique that can help detect and address biases within data, leading to improved learning outcomes. This method can prove to be essential for synthetic dataset services like the one used at the Clinical Practice Research Datalink in the UK. Using synthetic data instead of real patient data for complex statistical analyses, machine learning, and artificial intelligence (AI) research applications offers several advantages. Among them is the ability to detect and mitigate biases in the ground truth datasets, preventing synthetic data from being affected by structurally missing data or incorrect correlations and distributions found in the biased ground truth datasets. Various conventional techniques, including SMOTE and AdaSyn, enhance model performance by balancing classes. However, these methods can compromise fairness by equalising two classes through random sampling without considering the attributes. Fair-SMOTE deals with SMOTE and AdaSyn limits by balancing data based on class and sensitive attributes such that privileged and unprivileged groups have equal positive and negative examples in the data. While very useful for mitigating bias, as a side effect of improving fairness, this method produces data results that are not representative of real data distribution. BayesBoost has been shown to produce data that resemble the original data distribution, as observed in simulation experiment results by comparing ground truth data with BayesBoost results. The results obtained through the application of SMOTE, AdaSyn and Fair-SMOTE showed us comparable performance values, both for the results obtained on the CVD and Covid-19 data. The reported results show how the datasets resulting from the application of BayesBoost lead to better accuracy values than those obtained with SMOTE, AdaSyn and Fair-SMOTE. Additionally, preliminary experiments suggest that BayesBoost has the potential to improve other metrics beyond classification accuracy.

Based on the above, we offer three conclusions:

1. BayesBoost shows an excellent ability to identify under-represented groups within data given a sensitive attribute and a target disease
2. BayesBoost is able to handle this type of data bias by generating new synthetic data that do not deviate from the real data distribution
3. Additionally, BayesBoost improves learning performances

## 5 Further works

Although BayesBoost shows potential in detecting and correcting data biases in primary healthcare data, it has limitations. BayesBoost can tackle only one discrete sensitive attribute at a time, which may pose a challenge in scenarios with numerous sensitive attributes. Additionally, BayesBoost is best suited for categorical sensitive attributes and may be less beneficial for continuous or mixed-type data. This necessitates additional preprocessing steps for feature engineering when sensitive attributes are not categorical. Moreover, BayesBoost is primarily designed for binary disease classification, a choice made to enhance its effectiveness in correcting biases and improving predictive models for specific diseases. However, this specialization restricts its suitability for multi-label classification problems or situations that do not involve diseases. Furthermore, it is essential to recognize that BayesBoost's performance may be influenced by dataset-specific factors, computational resources, and domain-specific assumptions. Therefore, as we explore the potential to extend BayesBoost to handle non-discrete variables and assess biases in datasets with different structures, such as time series data, these limitations should be carefully considered and methodologically addressed. Future works will include further adaptation to broaden the scope of BayesBoost's applicability, allowing it to address biases associated with multiple sensitive attributes, data types, and multi-label classification challenges in primary healthcare data. The paper briefly introduced fairness metrics as an area for potential exploration for the generalisability of uncertainty analysis. In light of our findings, we plan to conduct further research to better understand the interactions between fairness metrics within our framework. Additionally, future efforts are directed towards optimizing parameters, such as p1 and p2, to enhance their generalisability and performance across diverse datasets.

## CRediT authorship contribution statement

**Barbara Draghi:** Writing – review & editing, Writing – original draft, Visualization, Validation, Software, Project administration, Methodology, Investigation, Formal analysis, Data curation, Conceptualization. **Zhenchen Wang:** Supervision, Software. **Puja Myles:** Supervision, Resources. **Allan Tucker:** Writing – review & editing, Supervision.

## Declaration of Competing Interest

The authors declare that they have no known competing financial interests or personal relationships that could have appeared to influence the work reported in this paper.

## Data Availability

The anonymised electronic healthcare record data used in this research is not publicly available but can be requested from CPRD subject to a data licence and research data governance (RDG) approval. The generated synthetic data set discussed in this paper can also be requested from CPRD subject to a data sharing agreement (DSA). Data access licence fees apply (https://cprd.com/data). **Code availability** All our R code is available via GitHub (https://github.com/barbaraDraghi/BayesBoost). The R package bnlearn (v4.8.1) is used for all Bayesian network inference.
